# Self-reported health and subsequent mortality: an analysis of 767 deaths from a large Thai cohort study

**DOI:** 10.1186/1471-2458-14-860

**Published:** 2014-08-20

**Authors:** Jiaying Zhao, Vasoontara Yiengprugsawan, Sam-ang Seubsman, Matthew Kelly, Chris Bain, Adrian Sleigh, The Thai Cohort Study Team

**Affiliations:** National Centre for Epidemiology and Population Health, Research School of Population Health, ANU College of Medicine, Biology and Environment, the Australian National University, Canberra, Australia; School of Human Ecology, Sukhothai Thammathirat Open University, Sukhothai, Thailand; QIMR Berghofer Medical Research Institute, Kragujevac, Queensland Australia

**Keywords:** Self-reported health, Cause-specific mortality, Thailand, Cohort study

## Abstract

**Background:**

Few studies have examined the link between self-reported health (SRH) and subsequent mortality in developing countries, and very few considered mortality effects of changes in SRH. We examined the relationship between SRH and subsequent all cause or cause-specific mortality in Thailand. We also noted if mortality varied after people changed their SRH.

**Methods:**

We used longitudinal data including SRH from a nationwide Thai Cohort Study (baseline 2005 - follow-up 2009) and linked to official death records (2005–2012). Cox regression examined the association between SRH in 2005 and subsequent all-cause mortality or cause-specific mortality, with results given as confounder-adjusted hazard ratios (HR). We further assessed association between changes in SRH during 2005–2009 and mortality from 2009 to 2012.

**Results:**

Poor SRH at baseline independently relates strongly with subsequent cardiovascular disease (CVD) mortality (HR = 2.8, CI: 1.3-5.9) and “other” causes of death (HR = 1.9, CI: 1.1-3.3) but moderately with cancer mortality (HR = 1.4, CI: 0.7-3.0). SRH did not exhibit a relationship with injury mortality (HR = 1.0, CI: 0.5-2.1). Worsening SRH from 2005 to 2009 associated with increased mortality in 2009–2012 for females but not for males.

**Conclusions:**

In Thailand, SRH is a good predictor of population mortality due to internal causes (e.g. CVD). SRH is holistic, simple to measure and low cost; when repeated it measures dynamic health status. In many developing countries chronic diseases are emerging and morbidity information is limited. SRH could help monitor such transitions in burdens and trends of population health.

## Background

Self-reported health (SRH) is a simple measurement of overall health. Potentially it could be used in developing countries to monitor changing health outcomes when information is scarce. In longitudinal studies of many high income countries self-reported health (SRH) was an independent predictor of subsequent mortality [1–3]. This SRH-mortality effect persisted after accounting for the influence of sociodemographic and medical risk factors [2], but the strength of the association differs by cause of death [4]. SRH associates with deaths due to heart disease, stroke, respiratory disease and cancer, but not with deaths due to accident and homicide [4, 5]. Because SRH approximates the holistic World Health Organization definition of health it fits well in many analyses of population health but interpretation of the actual risk and the underlying mechanisms are still being discussed [6].

Research on specific causes of death within cultures may help us understand mechanisms linking SRH and mortality [4, 5]. It would be worthwhile to investigate the SRH-mortality association in different cultural settings, especially in developing countries where information on this topic is limited. Culture and gender have influenced the degree of ill-health self-reported [1]. The cultural aspect operates partly through socio-economic status and there is some evidence that poorer people stoically report better health [7–11]. Poorer countries are likewise affected as shown in reports from India and China [12, 13]. Across cultures, gender effects are also widespread with women usually reporting worse health status [7, 9].

Culture and gender affect the strength of the SRH-mortality association [9, 11, 14, 15]. Also, people may change their SRH over time, and this may alter mortality risks [16, 17]. The link between poor SRH and mortality persists across various follow up periods (from 2 to more than 30 years) [2, 16]. But SRH is a continuous evaluation of our bodies and our health, so changes in SRH should be more predictive of mortality than a one-time assessment [3, 18, 19]. However, few population-level studies have examined change in SRH and its association with mortality [3, 16]. Even less is known about change in SRH and change in mortality in developing country settings.

Thailand is one such developing country in Southeast Asia and it is an informative setting to investigate SRH [20, 21]. Here we present the results of our longitudinal Thai study of the relationship between self-reported health (measured in 2005 and 2009) and subsequent all cause or cause-specific mortality (measured over the period of 2005–2012). We also noted if risk of dying varied after people changed their self-reported health. Results are adjusted for demographic characteristics, health behaviours, and an array of physical conditions.

## Methods

### Study population

Analyses were based on the Thai Cohort Study (TCS) of the health associations or consequences of socioeconomic development [20, 22]. Participants (N = 87,151) were distance-learning students (aged from 15 years to 87 years) enrolled at Sukhothai Thammathirat Open University (STOU), residing all over Thailand in 2005. Most STOU students remain embedded in their communities, work and families. This student body was chosen because their social geography, religion, and income are similar to the Thai population [20, 22]. TCS participants are well educated, an advantage for collecting self-reported information. TCS participants were followed up in 2009 by mail. Response was encouraged by telephone reminder. After 4 rounds of telephone contact (more than 100,000 phone calls) and 4 related mail-out rounds progressively over 16 months, 60,569 participants completed questionnaires and an overall response rate was 71% [22, 23].

TCS data were linked to the mortality register at the Ministry of Interior in Thailand using the citizen ID number which is unique for each Thai person. By August 15th 2012 there were 767 cohort deaths reported to the Ministry of Interior. The completeness of registration of adult deaths in Thailand was 86% from 1950–2000 [24]. Coverage improved further to 95% over the period reported here [25].

The Ministry of Public Health provided cause-of-death for all deaths occurring before the end of 2010 (N = 583). The vital statistics office at the Ministry of Public Health had investigated cause of death if it was ill-defined by investigating hospital records and performing verbal autopsies. After this adjustment, ill-defined deaths were only 8.4% (N = 49) of total deaths. Cause-of-death data were coded according to The International Statistical Classification of Diseases and Related Health Problem 10th Revision (ICD-10). There were 78 deaths from cardiovascular disease (CVD) (ICD code = I00-I99), 118 deaths from neoplasms (ICD code = C00-D48), 204 from injury (ICD code = V01-Y98), and 183 deaths from “other causes of death”.

### Measurements

Self-rated health during the past 4 weeks was assessed with a single standardised question “Overall, how would you rate your health over the last 4 weeks?”. This question is the first in the 8-item Short-Form Health Survey (SF8™) and was measured in both 2005 and 2009. Responses were categorized as excellent, very good, good, fair, poor, and very poor. For analyses, we combined responses that were categorically positive and those that were categorically negative. So we divided SRH into “excellent/very good/good/fair” versus “poor and very poor”, a division used in previous analyses of the data [7].

Analyses of SRH and mortality included independent variables measured at baseline (2005) - sex, birth year (equivalent to age group), and category of health insurance. In Thailand, health insurance is provided through three programs: the welfare system for civil servants, Social Security for private employees, and the Universal Coverage scheme available to Thai nationals which is known as the 30 baht scheme. In addition, some private hospitals are financed by patient self-payment and private insurance.

We analysed urban or rural residence and income using 2005 response in some analyses and 2009 response in other analyses (see below). Personal monthly income (Thai Baht) measured in 2005 was classified as “<=3000”, “3001 − 20000”, or “20001 and above”. Household income measured in 2009 was classified as “<7000”, “7001-30000”, or “> = 30001”.

Cohort questions on health behaviour emerged after pretesting and were adopted from available standards at the time. The process is described in Sleigh et al. [20] and Seubsman et al. [22, 23]. When possible we followed Thai standards set by the National Statistical Office. We assessed health behaviours (smoking, drinking, physical activities (PA)) in 2005 and 2009. Smoking in 2005 and 2009 was categorized into ‘never’ , ‘ex-smoker’ , or ‘current smoker’. Alcohol consumption in 2005 was classified as ‘occasional social drinker’ , ‘never’ , ‘current regular drinker’ or ‘now stopped’. In 2009, we revised the alcohol consumption classifications (advised by cohort members) as follows: ‘non-drinker’ , ‘light drinker (<=7 glasses per week)’ , or ‘moderate or heavy drinker (> = 8 glasses per week)’. Weekly physical activity in 2005 and 2009 was recorded as ‘less than 7 sessions’ or ‘7 sessions or more’ , according to a PA measure based on the International Physical Activity Questionnaire and the Active Australia Survey [26]. A session was defined as more than 20 minutes (mild, moderate or strenuous) or 10 minutes or more (walking) and the formula for estimating sessions per week was “2 × strenuous + moderate + mild + walking”.

We recorded doctor diagnosed ischemic heart disease, hypertension, cancer, or diabetes in 2005 and 2009, by using the question “have you ever been told by a doctor that you have any of the conditions listed below”. The accuracy of the data on hypertension was investigated by physician telephone interviews of a cohort sub-sample of 240 hypertensives and 240 normotensives [27]. Validation showed that the self-reported results for hypertension were acceptable (sensitivity 83%, specificity 71%, overall accuracy 75%). We also validated self-reported diabetes in 2009 and found 96% accuracy. We investigated self-reported height and weight and we have validated these measurements [28]. We calculated body mass index (BMI) in 2005 and 2009 using Asian BMI standards: underweight (<18.5), normal (18.5 - < 23), at risk (23 - <25), obese I (25 - <30), and obese II (> = 30) [26, 29]. We also questioned about injury history interfering with daily activities or requiring medical treatment in the preceding 12 months. We originally devised the injury question with advice from Monash Injury Research Institute, Monash University in Melbourne and we have used this question on baseline and follow-up surveys and injury publications of the Thai cohort since 2005.

### Statistical analyses

We examined the distribution of SRH in 2005 by sociodemographic attributes and by survival status on the 31st December 2010 and the 15th August 2012. Kaplan Meier survival curves display differential survival by baseline SRH (1st March 2005). End-point events were all-cause deaths until December 31st 2010, cause-specific deaths until December 31st 2010 (CVD deaths, cancer deaths, injury deaths, and all “other causes of death”), and all-cause deaths until August 15th 2012. The Log-rank test was used to assess the statistical probability of differential survival according to baseline SRH.

Multivariate hazard ratios (HR) and 95% CIs for mortality by baseline (2005) SRH were estimated using the Cox regression model. We confirmed proportionality of hazards by showing the good and poor SRH survival curves did not cross. Potential confounders included are known to associate with SRH and mortality [2, 14, 16]. We used Stata to test for collinearity of the independent variables using Variance Inflation Factor (VIF). We did not find any collinearity.

**Figure 1:**
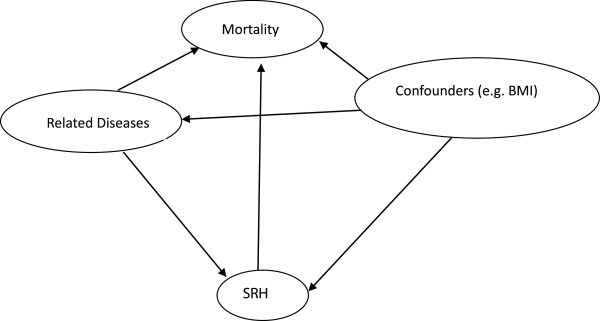
**Conceptual diagram of causal relationships among SRH, confounders, and mortality.**

We conceptualized the causal relationships in a figure depicting the independent effect of SRH on mortality along with the effect of confounders (Figure 1). This model draws on our earlier experience with these SRH data [7] and on insights and theories reviewed by Jylha [6]. We are aware that little is known of the mechanism linking SRH and mortality. However, in many other studies SRH has an independent effect detectable after adjustment for a wide array of confounders. In our study, we introduced the confounders into the all cause and cause-specific mortality models in three stages. First we estimated the association between SRH and mortality adjusted for birth year, gender, urban or rural residence (2005), monthly income (2005), and health insurance coverage, producing Model 1. We then adjusted Model 1 for health behaviours (smoking, drinking, and PA) in 2005, producing Model 2. Model 3 adjusted Model 2 for health conditions in 2005, including serious injury, BMI, and doctor diagnosis of ischemic heart disease, hypertension, cancer, or diabetes. The staged introduction of confounders allowed us to see directly their effects on mortality and the size of the study gave us the statistical power to include all confounders together in Model 3. Thus we were able to measure the survival effect of SRH on mortality in Model 3 which minimised confounding.

For each of four specific death outcomes (CVD, cancer, injury, and “all other causes of death”), three models were developed in the same way as for all-cause mortality. However, the 3 Models for cause-specific outcomes only include as explanatory variables diseases which are subsequently related to the cause-of-death outcome. For example, cancer morbidity was included as an explanatory variable in the model for cancer deaths, but not for CVD. For analysis of CVD mortality, we restricted explanatory morbidity to ischemic heart disease, hypertension, and diabetes, all of which are related to CVD. For all other causes of death, we restricted explanatory variables to diabetes as that death category included deaths due to diabetes. For injury death, we included serious injury among the explanatory variables.

Changes in SRH (2005–09) and all-cause mortality (2009–12) were analysed using Cox regression. SRH from 2005 to 2009 was classified into four categories: “unchanged good” (reference), “improved”, “worsened”, and “unchanged poor”. The end-point events for those who were followed up in 2009 were all-cause deaths until August 15th 2012. By that date in 2012, there had been 302 deaths among the 60569 participants of 2009 follow-up. Confounders include sex, birth year, place of residence (2009), household income (2009), health insurance coverage, smoking (2009), drinking (2009), PA (2009), BMI (2009), and doctor diagnosis of ischemic heart disease, hypertension, cancer, or diabetes in 2009. The analysis was further stratified by sex.

### Ethical approval

Informed written consent was provided by all participants, and ethics approval was obtained from Sukhothai Thammathirat Open University Research Committee and the Australian National University Human Research Ethics Committee.

## Results

At 2005 baseline, the proportion reporting poor health for males was lower than for females (3.8 vs. 5.2%, p < 0.005, see Table 1). The oldest group (born before 1960) had the lowest probability of reporting poor health (4.2%) and those born from 1975 to 1979 had the highest probability (5.1%). People with the lowest income had the highest proportion reporting poor health (5.3%), compared with middle income (4.5%) and the highest income (4.1%) groups (p < 0.005).

**Table 1:** **Socio-demographic attributes by self-reported health in the Thai cohort study, 2005**

Socio-demographic attributes	Self-reported health (SRH)*				
	Good		Poor		Total
	n	%**	n	%	n
Sex					
Males	37863	96.2	1497	3.8	39360
Females	45016	94.8	2489	5.2	47505
Birth year					
-1959	5160	96.1	207	3.9	5367
1960-1969	17215	95.8	755	4.2	17970
1970-1974	14543	95.5	687	4.5	15230
1975-1979	20816	94.9	1125	5.1	21941
1980-	25135	95.4	1212	4.6	26347
Personal monthly income (Baht)***					
<3000	8848	94.7	496	5.3	9344
3001-20000	63412	95.5	3019	4.5	66431
> = 20001	8561	95.9	367	4.1	8928
Total	82879	95.4	3986	4.6	86865

*SRH = self-reported health; six ordinal categories reduced to two ─ “good” and “poor”. “Good” included excellent, very good, good, and fair; “poor” included poor and very poor.

**Row percent.

***in 2009 35 Baht = US $1.

From baseline (2005) to 2012, people with poor SRH were more likely to die than those with good SRH (1.2% vs 0.9%, p < 0.05) (Table 2). Similarly, a higher mortality risk for people with poor SRH was observed until 2010 (p < 0.05). Cause-specific mortality analysis suggested that people with poor SRH were more likely to die from CVD (p < 0.05) than those with good SRH. But people with poor SRH had an almost equal risk of dying from injury as those with good SRH. There was a higher risk of dying from cancer and other causes of death for those with poor SRH compared to those with good SRH (p > 0.05).

**Figure 2:**
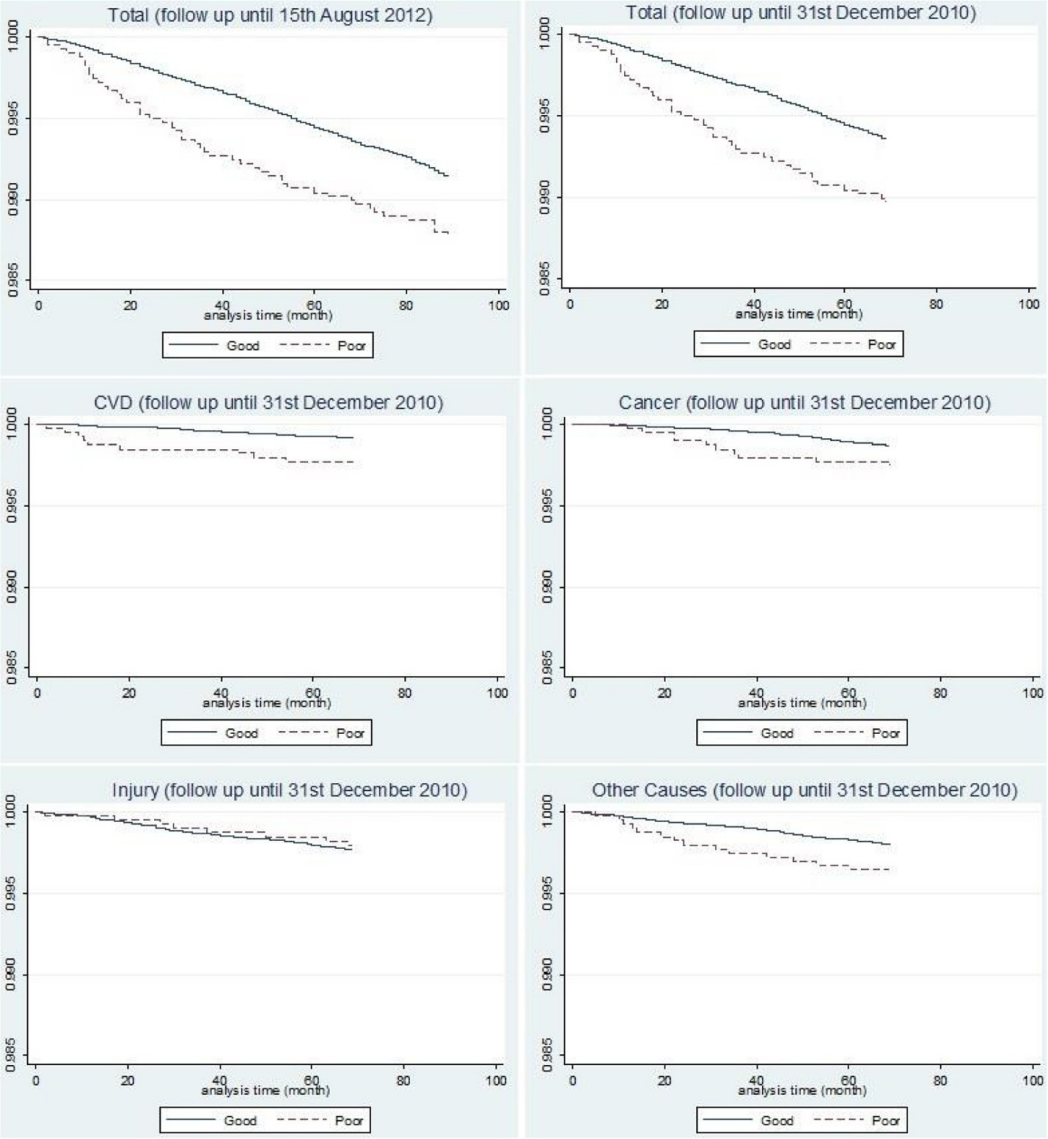
Kaplan-Meier survival curves for self-reported health (SRH) (good or poor)* by cause of death.

Kaplan-Meier survival curves for SRH (Figure 2) showed that avoiding mortality from overall deaths, CVD, cancer and other causes of death was less likely in those who initially rated their health as poor. The Log-rank test suggested that these differences in survival time were significantly different (p < 0.05). Injury mortality did not differ between those with good SRH and those with poor SRH.

**Table 2:** **Longitudinal survival status (2005–2012) by baseline (2005) self-reported health (SRH) for the Thai cohort study**

End-point for follow- up	Survival status	Self-reported health (SRH)*					
		Good		Poor		Total	
		n	%**	n	%**	n	%**
15th August 2012	Survived	82168	99.1	3937	98.8	86384	99.1
	Died	714	0.9	49	1.2	767	0.9
31st December 2010	Survived	82343	99.4	3945	99.0	86568	99.3
	Died	539	0.7	41	1.0	583	0.7
	CVD deaths	69	0.1	9	0.2	78	0.1
	Cancer deaths	108	0.1	10	0.3	118	0.1
	Injury deaths	194	0.2	8	0.2	204	0.2
	Other deaths	168	0.2	14	0.4	183	0.2

*SRH = self-reported health; six ordinal categories reduced to two ─ “good” and “poor”. “Good” included excellent, very good, good, and fair; “poor” included poor and very poor.

**Column percent.

In the Cox proportional hazard model, those reporting poor health at baseline, after controlling for age, gender, rural or urban residence in 2005, monthly income, and category of health insurance, had a significantly higher risk of subsequent death (both till 2010 and till 2012) (Model 1) (Table 3). Further adjustment for behaviour factors (smoking, drinking, and PA) strengthen these associations (Model 2). Finally, the addition of diagnosed diseases and BMI attenuated these associations, but patterns remained similar and significant (Model 3). The hazard ratios were higher in the short term (HR = 1.6, p < 0.05 during the follow up till 2010) than in the long-term (HR = 1.5, p < 0.05 during the follow up till 2012).

**Table 3:** **Mortality and poor self-reported heath (SRH)** ^†^ **in the Thai cohort study, 2005-2012**

Death category	Mortality hazard ratios (95% CI) for poor SRH in 2005		
	Model1	Model 2	Model 3
All-causes (till August 2012, 722 deaths)	1.55 [1.15-2.09]*	1.57 [1.17-2.13]***	1.45 [1.07-1.96]*
All causes (till December 2010, 549 deaths)	1.69 [1.22-2.36]***	1.74 [1.25-2.42]***	1.59 [1.14-2.22]**
CVD (72 deaths)	2.95 [1.41-6.17]***	3.10 [1.47-6.51]***	2.78 [1.31-5.93]**
Cancer (111 deaths)	1.70 [0.83-3.50]	1.65 [0.80-3.41]	1.44 [0.69-3.01]
Injury (194 deaths)	1.00 [0.49-2.03]	1.08 [0.53-2.19]	1.03 [0.50-2.11]
Others (174 deaths)	1.97 [1.13-3.40]*	1.98 [1.14-3.44]*	1.92 [1.10-3.31]*

^†^SRH = self-reported health; six ordinal categories reduced to two ─ “good” and “poor”. “Good” included excellent, very good, good, and fair; “poor” included poor and very poor.

*p < 0.05, **p < 0.01, ***p < 0.005; hazard ratios compare mortality over the follow-up period by initial SRH (reference category = “good” at baseline in 2005).

Note: the three models were constructed as follows:

Model 1 adjusted for sex, birth year, monthly income, urban or rural residence in 2005, and categories of health insurance.

Model 2: Model 1 + behaviour factors (smoking, drinking, and PA).

Model 3: Model 2 + BMI + related diseases reported in 2005 baseline; For total mortality, model included ischemic heart disease, hypertension, cancer, diabetes, and serious injury; For CVD mortality, model include ischemic heart disease, hypertension, and diabetes; For cancer mortality, model included cancer; for injury mortality, model included serious injury; for other causes of death, model included diabetes.

Cause of death analysis showed that SRH strongly predicted mortality from CVD (HR = 2.8, p < 0.005) after adjusting for demographic factors, lifestyle variables, BMI, and related doctor-diagnosed diseases (ischemic heart disease, hypertension, and diabetes). Respondents reporting poor health were almost twice as likely to die from “other causes” in the follow-up (HR = 1.9, p < 0.05) after full adjustment for the covariates (Model 3). People with poor SRH at baseline had a modest but non-significant increase in cancer mortality (HR = 1.4, p > 0.05). This was different to the relationship between SRH and injury, which showed no difference in mortality risk between poor SRH and good SRH (HR = 1.0, p > 0.05).

SRH change over the first period (2005–09) influenced survival over the following period (2009–12) (Table 4). People who died from 2009 to 2012 were more likely to report worsening SRH from 2005 to 2009 (7.2%) than those who survived (4.4%); when compared by two-tailed Z test these proportions were significantly different (Z = 2.1, p < 0.05). After full adjustment for demographic characteristics, risk behaviours, and self-reported doctor-diagnosis of certain diseases in 2009, people who reported worsening SRH between 2005 and 2009 experienced higher but non-significant subsequent mortality hazard during the following period 2009 to 2012 (HR = 1.6, p = 0.07). However, among males, there was no tendency of increased risk for those reporting worsening health. In contrast, among females, subsequent mortality risk for those reporting worsening SRH between 2005 and 2009 was significantly higher than those with unchanged good SRH (HR = 2.7, p < 0.05). Females reporting improved SRH and unchanged poor SRH also had higher risks of mortality than those reporting unchanged good SRH, though these increases were not statistically significant.

**Table 4:** **Longitudinal outcomes (survival and mortality 2009–2012) by change in self-reported health (SRH)** ^**†**^ **in the Thai cohort study, 2005-2009**

Change in self-reported health 2005–09 (ΔSRH)	2012 Survival status (%) by ΔSRH ^††^		Hazard ratios (95% CI) for mortality from 2009 to 2012 by ΔSRH		
	Survived (n = 52859)	Died (n = 247)	Total (n = 247)	Males (n = 164)	Females (n = 83)
All good	91.1	87.6	Ref	Ref	Ref
Better	3.5	3.2	0.86 [0.42-1.77]	0.33 [0.08-1.34]	1.94 [0.83-4.50]
Worse	4.4	7.2	1.57 [0.96-2.56]	0.96 [0.44-2.06]	2.70 [1.41-5.20]***
All poor	1.0	2.0	1.50 [0.60-3.70]	1.07 [0.26-4.46]	2.18 [0.63-7.62]

^†^SRH = self-reported health; six ordinal categories reduced to two ─ “good” and “poor”. “Good” included excellent, very good, good, and fair; “poor” included poor and very poor.

^††^Column percentage.

p < 0.05, p < 0.01, ***p < 0.005; hazard ratios compare mortality over the follow-up period by change in SRH over the previous period. All three models include age category (5 groups), household income in 2009 (3 categories), health insurance status in 2005, urban or rural residence in 2009, smoking (2009), drinking (2009), physical activities (2009), ischemic heart disease (2009), cancer (2009), hypertension (2009), and diabetes (2009); for total population, models include sex as well.

## Discussion

Among Thai cohort members, baseline SRH (2005) related to subsequent mortality risk over the next five or seven years, after controlling for socio-demographic factors, health behaviours, and baseline physical conditions. Furthermore, cause-specific analysis revealed the SRH relationship was strong for subsequent CVD mortality (HR = 2.8) and “other causes of death” (HR = 1.9). Cancer mortality among people with poor SRH increased moderately but not significantly (HR = 1.4). SRH did not predict subsequent injury mortality (HR = 1.0). Worsening SRH over time increased subsequent mortality among females but not for males.

Other studies also found that self-reported health is a predictor of subsequent adult mortality risk and support the use of this global measure of health status in developing settings [1, 12, 13]. However, developing country information on SRH and mortality is still quite limited. Also, in such settings, complete and accurate data on cause specific mortality are uncommon. Additionally, SRH does not predict all causes of mortality equally in our data, consistent with previous research [4, 5]. Benjamins et al. [4] speculated that the stronger relationship between SRH and CVD mortality or certain other causes of death (e.g. diabetes, respiratory and infectious diseases) may be because these diseases have more symptoms and treatments, thus leading to greater departure from good overall health. In contrast, as injury is often unexpected and sudden, and unconnected with previous physical health status, a relationship between SRH and injury mortality is not evident.

SRH evaluates health status dynamically [19]. Across time, proximate SRH is a more effective predictor for subsequent mortality. Our results are consistent with previous findings that hazard ratios for SRH tend to be higher across a short-term period and lower across a long-term period [30]. A reasonable explanation relates to the higher predictive power linking recent SRH and immediate survival [3]. This is consistent with previous findings in Western populations [3, 16, 31].

Evidence diverges on gender differences in trends of self-reported-health and the relationship to all cause mortality [2, 32–34]. We found that worsening SRH (2005–2009) has a higher hazard ratio for subsequent all cause mortality (2009–2012) among females than males. This could have resulted from the higher proportion of injury deaths (which have no association with SRH) among males. For example, in our 2005–2010 data, the proportion of injury deaths is larger for males (38%) than for females (29%) in our young cohort. Some previous research has shown that men have stronger associations between SRH and mortality [32, 35, 36]. Those reports are based on elderly populations which have lower rates of injury than their younger counterparts. So the results for males are as expected.

Another interpretation is that cultural differences may mean that Thai females are more sensitive to changes in SRH than Thai males. In support of that theory, we found that the death hazard ratio for “worse SRH” was stronger among females (2.7, CI: 1.4-5.2) than males (1.0, CI: 0.4-2.1). This situation adds to our early finding (TCS, 2005 baseline) showing that females were relatively unaffected by income or education when reporting SRH, a pattern that differed from males and which could be culturally constructed [7]. We have no further evidence bearing on this issue of gender differences with SRH, but our mortality data suggest it would be a fruitful topic for future research.

This study is one of the first attempts to link SRH with mortality data, taking cause of death into account in developing settings. National cause-of-death data in Thailand ten years ago had a high proportion of ill-defined deaths [37]. However, in our study, after investigations by the Ministry of Public Health, ill-defined deaths were reduced to 8.4% of total deaths and this decreased a major source of error. Our analyses are based on a large national longitudinal cohort and capture information on changes in SRH and association with mortality. Similar analyses are reported for developed countries but are limited in developing countries [16–18]. One notable advantage of our study relates to cohort members as health and health-risk informants. They are distance-learning university students embedded in their communities and are accustomed to education by mail. As they are well educated, we expected good quality information on exposures and diseases [38]. We found that they are particularly capable of providing complex data about themselves, their environment, and their health, as shown by the stream of information that has emerged on the Thai health risk transition, including many publications and presentations on this topic [21, 26–28]. As part of this research, we have validated many of the self-reports for diseases or exposures. General information and references for these salient features of the Thai Cohort Study are given in the Methods.

Some limitations of the study should be borne in mind. For example, we have made repeat measures of important variables and were not always able to use identical questions. Thus, we changed the question on drinking in response to our experience of the baseline study and the need to identify binge drinkers in the 2009 follow-up survey. But we devised the revised questions so that the data from 2005 and 2009 could be manipulated and harmonized before analysis. It was not always necessary to use a repeat measure of drinking and in this report the 2005 baseline data were used for initial assessments and 2009 data were used for dynamic assessments of changing SRH.

There were other study features which could become limitations. First, confounders (e.g. smoking, doctor diagnosed diseases) included in the analyses are based on self-reported information. However, we have validated many of these variables as described in the Methods and we discuss above the advantage of having university student informants. Second, as cause-of-death data are only available till December 2010, we did not analyse the effect of changes in SRH from 2005 to 2009 on specific causes of death for the subsequent period (2009–12). As the cohort ages more information will emerge. Third, our cohort members are still young and mortality rates are low; this restricted our analytical power but will also resolve with the passage of time. Fourth, the classification of 2005 SRH into “excellent/very good/good/ fair” versus “poor/very poor” may reduce statistical power as the proportion of “poor/very poor” SRH was low (3.8% for males; 5.2% for females). It would be possible to balance the categories somewhat by including fair with “poor/very poor” but “fair” SRH is reasonably classified linguistically as it is (ie. with the positive group) because it is likely to be understood as “normal” health status in the Thai language [6].^.^ Also, there are no statistical differences in percent probability of dying among the four positive categories (excellent/very good/good/fair) (0.76%, 0.93%, 0.85%, 0.85%); but negative categories (poor/very poor) were more likely to die (1.21%, 1.39%) and when negative categories combined are compared to positive categories combined the difference in percent probability (0.86% vs 1.23%) is quite significant (p = 0.015). Thus positive and negative categories are reasonably categorized as “good” and “poor”. It is possible that people with poor SRH may be an outlier group (e.g. poor mental health status) having higher mortality risks. However, the mechanism of the relationship between poor SRH and mortality is beyond the scope of this research and it could be studied further in the future.

In future, as the cohort follow-up continues we will be able to enhance our findings as cohort members get older and we gather more information on SRH. In addition, we may examine the association between changes in SRH and specific cause of death when cause-of-death data from 2010 become available. More analysis is needed to improve understanding of the gender differentials in the relationship between changing SRH and mortality, especially by causes of death in young age groups.

## Conclusions

In conclusion, among members of the Thai Cohort Study, SRH is not a good predictor of external causes of death. But the hazard ratios revealed that SRH is a good predictor of most internal causes of death (e.g. CVD). Indeed, SRH trends are useful for predicting trends in emerging causes of deaths relating to a health-risk transition because internal causes of deaths are responsible for most of these trends. Accordingly, when mortality was analysed against SRH changes over four years (2005–2009), all cause mortality rose and fell in parallel to SRH changes. This highlights the value of SRH to reveal the dynamics of population health in developing countries where chronic diseases are emerging and information on morbidity is still limited. Furthermore, SRH has other interesting properties: it is an holistic measurement that can be established easily at low cost and it can be followed through time.

## Abbreviations

BMI: Body mass index
CI: Confidence interval
CVD: Cardiovascular disease
HR: Hazard ratios
ICD-10: The International Statistical Classification of Diseases and Related Health Problems 10th Revision
NHMRC: National Health and Medical Research Council
PA: Physical activities
SF8™: The 8-item Short-Form Health Survey
SRH: Self-reported health
STOU: Sukhothai Thammathirat Open University
TCS: Thai Cohort Study.
